# Exploring Flooding Challenges, Causes, and Mitigation Strategies in Rice

**DOI:** 10.1155/ijog/7576298

**Published:** 2026-04-03

**Authors:** Abdoul-Razak Oumarou Mahamane, Junior S. Kamara, Moise Hubert Byiringiro, Alpha Sow

**Affiliations:** ^1^ Département des Cultures Irriguées, Institut National de la Recherche Agronomique du Niger, BP 429, Niamey, Niger; ^2^ Crop Resource Division, Ministry of Agriculture, Ellen Johnson Sirleaf Ministerial Complex, P. O. Box 10-90100, Monrovia, agriculture.tn, Liberia; ^3^ Rwanda Institute for Conservation Agriculture (RICA), Kagasa-Rweru Rd off RN 15 P.O.Box 017 Nyamata, Bugesera, Rwanda; ^4^ International Maize and Wheat Improvement Center (CIMMYT-SENEGAL), ISRA/CERAAS, P.O.Box.3320, Thiès, Senegal

## Abstract

Flooding is a dominant constraint to rice production in lowland and floodplain ecosystems, where stress intensity, timing, and duration vary widely across agroecological zones. This review synthesizes current knowledge on flooding typologies affecting rice, including anaerobic germination failure, flash flooding, stagnant flooding, and deep‐water submergence, and critically examines how rice plants respond at physiological, anatomical, and molecular levels. We integrate evidence on key adaptive mechanisms, including anaerobic metabolism during germination, SUB1‐mediated quiescence under short‐term submergence, SNORKEL‐driven internode elongation in deep‐water environments, aerenchyma development, leaf gas films, and antioxidant defense systems. Particular emphasis is placed on the genetic architecture underpinning these responses, highlighting the successes and limitations of the SUB1 locus and the necessity of multitrait pyramiding to address prolonged, deep, or sequential flooding events. Drawing on case studies from Africa and Asia, the review demonstrates that flooding impacts are strongly context‐specific, shaped by rainfall variability, hydrology, soil properties, and water management infrastructure, with disproportionate socioeconomic consequences for smallholder farmers. We argue that reliance on single‐gene solutions is insufficient under climate change scenarios that intensify flood unpredictability. Instead, resilient rice systems require an integrated strategy combining stress‐tolerant varieties, adaptive crop and nutrient management, and climate‐informed decision‐making. By consolidating physiological insights, breeding advances, and regional flood dynamics, this review provides a targeted framework for developing rice varieties and management options tailored to diverse flooding regimes, with direct implications for food security in flood‐prone regions.

## 1. Introduction

Rice is a critical staple food crop, serving as the main source of calories for over half of the global population. Approximately 40 million hectares of lowland regions worldwide are dedicated to rice cultivation [1]. Its production plays a central role in supporting the livelihoods of millions of smallholder farmers. Despite its importance, rice farming is highly vulnerable to environmental hazards, particularly flooding, which is considered one of the most hazardous natural disasters and significantly restricts rice cultivation in various regions around the globe [1, 2]. Each year, more than 100 million rice farmers are affected by unfavorable flooding due to submergence [3].

Food security is defined as a situation that exists when all people, at all times, have physical, social, and economic access to sufficient, safe, and nutritious food that meets their dietary needs and food preferences for an active and healthy life [4]. It encompasses multiple dimensions, including the availability of food, the ability to access and utilize it, its stability over time, and aspects of empowerment and sustainability [5]. Rice contributes directly to these dimensions, and disruptions to its production have immediate and far‐reaching consequences for household and national food security.

Climate change poses a serious threat to food security by influencing agricultural productivity, particularly in tropical regions where farming is highly dependent on climatic conditions [6]. Its effects are exacerbated by smallholder farmers’ limited capacity to anticipate or adapt to changes, leaving them vulnerable to extreme events. Predicted climate impacts include flooding, droughts, dryness, and altered rainfall patterns, all of which threaten crop productivity [7]. In particular, flooding is expected to increase in both depth and volume by up to 60%, potentially causing agricultural damage to rise by 2–6 times, with corresponding economic losses in river basins projected to escalate between 2 and 38 times [8].

Flooding has direct and severe impacts on rice production. It can cause two distinct types of stress: submergence stress, which occurs when the entire plant is submerged underwater, and waterlogging stress, which occurs when only parts of the leaves and stems are submerged [9]. Flooding is a complex stress that primarily limits the supply of oxygen (O_2_) and causes accumulation of carbon dioxide (CO_2_) within plant tissues due to the slow movement of gases in water. Reduced molecular oxygen leads to lower ATP production and limits carbohydrate availability, which significantly impacts plant growth and survival [10]. The economic consequences of such events are substantial; for example, in 2018, flooding in the Long Xuyen Quadrangle of Vietnam’s Mekong Delta resulted in economic losses of approximately 121 million USD, representing around 1.47% of the region’s gross regional domestic product ([11]; Table 1).

**TABLE 1:** Socioeconomic impacts of flooding on rice cultivation.

Country/region	Yield loss	Socioeconomic impact	Source
Tanzania (Lower Rufiji)	75%–100% yield loss	Farm abandonment, land sales, migration	[12]
Benin	Significant yield reduction	Correlation between flooded land and yield loss	[13]
Bangladesh	Up to 10% yield loss	Price spikes, food insecurity	[14]
Mekong subregion	47% of households with heavy crop losses	Household debt, limited government aid	[15, 16]
China	5.3% (∼10 Mt per event)	∼USD 24 million loss per flood event	[17]
East China (Yangtze Basin)	Up to −14%	Economic losses up to ∼USD 57 million per flood event	[17]
India	−6.4% (∼5 Mt per event)	∼USD 29 million loss per flood event	[17]
Northeast and Eastern India	Up to −7%	Economic losses up to ∼USD 32 million per event; strong livelihood impacts	[17]
Southeast Asia	Country losses 2.6%–4.4%	Severe livelihood disruption	[17]

Given these challenges, adapting farming practices to climate change requires a comprehensive understanding of how environmental changes affect the livelihoods of farming families [11]. Therefore, there is a critical need to examine the impacts of climate change on rice production at both global and local scales to develop effective adaptation strategies. In this review, we explore the physiological, genetic, and economic adaptations that enhance rice resilience to climate‐driven flooding. We highlight key adaptive traits in flooded rice varieties and provide an updated overview of the molecular genetics and mechanisms underlying submergence tolerance.

## 2. Types of Flooding Stress in Rice Ecosystems

### 2.1. Flooding During Germination

Soil waterlogging typically occurs after it rains postseeding, especially in fields that are not evenly leveled. The germination of seeds under flooded conditions is termed anaerobic germination (AG). Flooded conditions generate hypoxic environments that limit oxygen availability, resulting in slow germination and irregular or delayed seedling growth. These challenges, combined with increased weed pressure, hinder the widespread adoption of direct‐seeded rice in flood‐prone regions [18]. The majority of rice varieties are susceptible to flooding, which can reduce their growth at the germination stage [19]. AG is a complex trait regulated by multiple genes involved in essential processes, including starch degradation, glycolysis, fermentation, and various other metabolic and biochemical activities [18]. Therefore, AG plays a crucial role in ensuring uniform germination and improving crop establishment in flooded conditions [20].

### 2.2. Flash Flooding

Flash flooding can occur when heavy rains or rivers and streams overflow, often lasting for a short period of 1–2 weeks. This can lead to plants being completely submerged [21]. Lands in low‐lying areas, especially those near streams and rivers, often experience flash flooding [1]. Although flash flooding is usually not deep, it is the most destructive type because it can occur multiple times within a single season and cause yield losses of up to 80% in rice production [1, 22]. Flash flooding tolerance is the ability of certain rice varieties to endure submersion in water for 1–2 weeks and then resume growth once the water recedes [23].

### 2.3. Stagnant Flooding

In stagnant flooding conditions, water levels can rise between 25 and 50 cm, with some parts of the rice plants still visible above the water. Extended water accumulation can last from a few weeks to several months, especially in areas without effective drainage systems [24]. This issue is particularly prevalent during the monsoon season, when heavy rainfall causes water channels to overflow, especially near rivers. Stagnant flooding greatly reduces rice production, causing yield losses of up to 83%, even when the plants are not completely covered by water [25]. Stagnant flooding reduces yield by decreasing tillering, panicle size, and spikelet fertility and increases lodging in genotypes with high elongation ability [26, 27]. The effects of stagnant flooding are more serious when it follows a period of complete submergence, which often happens in flood‐prone areas [28].

### 2.4. Deep‐Water Flooding

In deep‐water flooding, water can stay in one place for an extended period. Occasionally, the level can reach heights of up to 4 m [19]. Flooding stress can persist for months, influenced by the land’s features and weather conditions. Rice plants that are adapted to these conditions speed up their growth in response to rising water levels to prevent being completely submerged [1]. These deep‐water rice varieties avoid total flooding by elongating their stems rapidly, a process that uses a significant amount of carbohydrates. It can grow as much as 25 cm each day as the water level rises and can attain a height of 5 m with its panicle and top leaves under these conditions [19].

## 3. Geophysical and Climatic Determinants of Flood Vulnerability in Rice Cultivation

The vulnerability of rice fields to flooding is influenced by a combination of geographical, hydrological, and climatic factors (Table 2). The geophysical dimensions of flood impacts on rice fields are shaped by a complex interplay of climate, hydrology, topography, soil, and infrastructure. These factors determine the likelihood and severity of flooding and guide the development of effective adaptation and risk reduction strategies for rice‐based systems [12]. In recent decades in Africa, we have observed a marked intensification and erratic shift in rainfall patterns across many traditional rice‐growing regions. This shift has changed previously secure fertile land into a flood‐prone zone, with rainfall becoming both more severe and less predictable, posing a significant threat to rice production and food security [13].

**TABLE 2:** Key drivers of rice field vulnerability to flooding.

Factor	Mechanism	Affected rice ecologies and regions in Africa	Source
Changing rainfall patterns	Increased rainfall intensity and variability lead to more frequent flash floods and prolonged inundation in rainfed systems	Rainfed lowland rice systems across Sub‐Saharan Africa, particularly West and East Africa	[29]
Poor drainage	Inadequate drainage in inland valley bottoms causes prolonged waterlogging and crop loss	Inland valley rice systems of West Africa (e.g., Nigeria, Benin, Côte d’Ivoire)	[13]
Topography	Low‐lying inland valleys and flood‐prone rainfed lowlands accumulate floodwaters	Flood‐prone rainfed lowlands and inland valleys across Sub‐Saharan Africa	[12]
Soil type	Heavy clay soils prolong inundation; sandy soils increase variability between drought and flooding	Inland valley bottoms and lowland rice ecologies in West and Central Africa	[12]
Hydrology	River overflow and high groundwater levels contribute to field inundation	Major African river basins supporting lowland rice (Niger, Senegal, Volta, Zambezi)	[15]
Climatic patterns	Climate change amplifies extreme rainfall events, increasing flood frequency and intensity	Rainfed rice systems across Sub‐Saharan Africa	[29]

The variability in seasonal rainfall, including both droughts and floods, has decreased rice productivity and increased vulnerability among smallholder farmers [29]. In Zambia, for instance, such variability has had a prominent impact on agricultural productivity, underlining the need for adaptive strategies to mitigate these adverse effects [30]. Additionally, poorly designed or maintained drainage systems in rice fields intensify flood impacts, resulting in prolonged waterlogging, root suffocation, and crop failure [31].

In West Africa, the absence of flexible and sustainable water control structures has been identified as a key factor intensifying flood damage [31]. Effective water management is essential for mitigating flood risk and navigating dry spells, as improper drainage can create a cycle of alternating stressors that undermine rice yield. This challenge is prevalent in regions with heavy clay soils, which have low permeability and retain water for extended periods, increasing the duration and severity of flooding [31]. Inversely, sandy soils offer rapid drainage; however, they fail to retain sufficient moisture during dry spells, leading to water stress [12].

In West Africa, flexible and sustainable water control structures are necessary to manage both floods and dry spells. Improper drainage can intensify flood damage, making it crucial to implement effective water management practices [13]. In regions like Burkina Faso, improved water control and drainage infrastructure are essential for sustaining rice production in the face of increasing flood frequency [13]. Rice fields situated in low‐lying plains and floodplains are inherently more susceptible to flooding due to their landscape position. These areas naturally accumulate floodwaters, and their proximity to rivers or deltas further amplifies exposure during high‐flow events [32]. In Tanzania, for instance, the lower Rufiji floodplain is prone to extensive flooding due to its downstream location and the convergence of multiple sub‐basins. The spatial clustering of rice cultivation in such regions is both a historical adaptation to water needs and a present‐day source of risk [12].

The hydrological regime, including the river flow and groundwater levels, directly influences flood risk in rice cultivation areas. River overflow and high groundwater levels contribute to field inundation, making it essential to manage water levels effectively, particularly when combined with intense rainfall. In regions like the Mekong Delta, the hydrological dynamics play a key role in determining flood risk and agricultural productivity [29]. Climate models project that African rice‐growing regions, particularly those in floodplains, will experience more frequent and intense rainfall extremes. This is expected to increase the incidence and severity of floods, with direct implications for rice production [29]. In Casamance, Southern Senegal, regional climate models indicate that extreme precipitation events are likely to coincide with critical rice growth phases, significantly raising the risk of yield loss and menacing food security [33]. Similarly, in Ghana’s Upper East region, flood events have been shown to significantly reduce rice yields and compromise food security. This situation is exacerbated by pervasive poverty and weak infrastructure, which together magnify the vulnerability of farming households [34].

## 4. Physiological and Molecular Mechanisms of Flood Survival

### 4.1. Physiological Mechanisms

Rice plants have developed specialized physiological strategies for flood survival that correlate with the type, duration, and depth of flooding challenges: (a) the quiescence (*hold-your-breath*) strategy associated with submergence tolerance; (b) the escape (snorkel) strategy for deep‐water tolerance; and (c) anatomical adaptations like aerenchyma formation [35].

Several studies have been carried out on flood‐tolerant rice genotypes to understand the mechanisms of adaptation to flood constraints, which are due to oxygen shortage during floods causing hypoxic or anoxic states [19]. Landraces and cultivars have developed the quiescence strategy, mediated by key genetic factors like the SUB1A gene. When submerged, these plants suppress shoot elongation and metabolic activity, conserve carbohydrates and chlorophyll, and enter a dormancy‐like state until floodwaters recede [19].

At the germination stage, tolerant genotypes rely primarily on physiological mechanisms rather than structural changes, including a rapid shift to anaerobic metabolism and strong activation of fermentative pathways. Enhanced α‐amylase activity promotes starch breakdown, ensuring a continuous supply of soluble sugars for ATP production under oxygen deficiency [36]. Rapid coleoptile elongation, supported by expansin gene induction, improves access to oxygen near the water surface and enhances seedling emergence under flooded conditions [37]. Regulation of carbohydrate signaling through CIPK15 and OsTPP7 further supports energy balance during AG [38, 39].

Physiologically, quiescent varieties exhibit minimal shoot elongation during submergence, high retention of nonstructural carbohydrates, maintenance of chlorophyll in submerged leaves, and reduced consumption of stored energy [35]. Suppression of gibberellin‐induced growth by the SUB1A gene is fundamental, as excessive elongation depletes critical carbohydrate reserves and impairs recovery. Increased activity of fermentative enzymes, including alcohol dehydrogenase (ADH) and pyruvate decarboxylase (PDC), supports anaerobic ATP generation during oxygen scarcity, further aiding survival [40].

Rice cultivated in regions subjected to rising floodwaters for many weeks or months utilizes an escape mechanism identified by rapid internode elongation, allowing leaves to remain above the water surface [41]. In deep‐water rice, internode elongation rates can reach up to 25 cm per day under suitable conditions [19]. The escape response is primarily triggered by ethylene accumulation under submergence, which increases the biosynthesis and sensitivity of gibberellin and reduces abscisic acid levels. This hormonal interplay improves cell division and elongation, enabling swift shoot growth to restore access to light and atmospheric gases. The formation of adventitious roots at upper stem nodes is another important adaptation, which enhances oxygen and nutrient uptake from aerated water layers when soil roots are impaired [19].

Moreover, the key anatomical adaptation under stagnant flooding is the formation of aerenchyma, an air‐filled cavity within the root and shoot cortex. Aerenchyma is produced mainly through cell death (lysigenous aerenchyma), regulated by ethylene and reactive oxygen species (ROS) [42]. This adaptation provides low‐resistance internal pathways for the diffusion of gases from shoots to submerged roots, supporting aerobic respiration even in oxygen‐depleted soils [43]. Furthermore, the development of a barrier to radial oxygen loss (ROL) in the basal root zones minimizes oxygen leakage and enhances the rice plant’s internal aeration, contributing to root survival under prolonged submergence [35].

These differential responses highlight the necessity of stage‐specific breeding strategies: Selecting for AG traits enhances early‐stage survival, whereas incorporation of SUB1 and elongation‐related quantitative trait loci (QTLs) improves tolerance during vegetative and stagnant flood conditions [23, 44]. Integrating these physiological and morphological traits into breeding programs provides a holistic approach to developing rice varieties capable of withstanding the diverse flooding stresses encountered in rainfed lowlands.

### 4.2. Role of Antioxidant Enzymes and Leaf Gas Films in Submergence Tolerance of Rice

Complete submergence imposes multiple constraints on rice plants by limiting gas diffusion, reducing light availability, and inducing hypoxic or anoxic conditions in submerged tissues [45, 46]. One of the major physiological consequences of submergence is oxidative stress, which arises both during flooding and, more severely, during post‐submergence re‐aeration, when rapid re‐oxygenation enhances ROS production [47]. Rice leaves possess superhydrophobic epidermal surfaces that retain a thin layer of gas, known as a leaf gas film, upon complete submergence [46, 48]. These gas films substantially reduce resistance to gas diffusion at the leaf‐water interface, thereby enhancing the diffusion of oxygen and carbon dioxide between floodwater and leaf tissues [48, 49]. Using microsensor measurements and three‐dimensional diffusion–reaction modeling, Verboven et al. [48] demonstrated that the presence of a leaf gas film markedly increases internal leaf oxygen partial pressure under submergence, even when ambient oxygen levels in floodwater are low. Importantly, gas films remain effective in sustaining internal oxygen status even when stomatal conductance is minimal, indicating that gas exchange can proceed via the gas film‐stomatal pathway despite partial stomatal closure [48]. Leaf gas films support underwater photosynthesis and aerobic respiration, particularly under stagnant or nocturnal conditions when photosynthetic oxygen production is limited by maintaining internal aeration [48, 49]. The functional importance of leaf gas films for submergence tolerance has also been confirmed under field‐relevant conditions, including saline floodwaters [50].

Submergence and subsequent re‐aeration induce oxidative stress in rice through enhanced ROS generation, leading to chlorophyll degradation, lipid peroxidation, and inhibition of photosynthetic enzymes [47]. Comparative studies of rice cultivars differing in submergence tolerance have shown that tolerant genotypes, such as FR13A and Sub1‐introgressed lines, exhibit significantly higher activities of antioxidant enzymes, including superoxide dismutase, catalase, ascorbate peroxidase, guaiacol peroxidase, glutathione reductase, and dehydroascorbate reductase, during both submergence and post‐submergence recovery [47]. In addition to enzymatic antioxidants, tolerant cultivars maintain higher levels of non‐enzymatic antioxidants, particularly ascorbate, which plays a central role in detoxifying hydrogen peroxide and preserving photosynthetic integrity [47]. Positive correlations between antioxidant enzyme activities, chlorophyll content, Rubisco activity, and net photosynthetic rate indicate that an efficient antioxidant system contributes directly to the protection of the photosynthetic apparatus under submergence stress [47]. Although leaf gas films do not directly scavenge ROS, their role in improving internal oxygen availability indirectly moderates oxidative stress by reducing the severity of hypoxia and limiting abrupt hypoxia–reoxygenation transitions during de‐submergence [45, 48]. This buffering effect likely reduces oxidative damage and facilitates more effective operation of antioxidant defense systems during recovery. Agronomic and environmental factors that enhance underwater light penetration, such as wider plant spacing, further strengthen this integrated tolerance mechanism by delaying leaf senescence, slowing carbohydrate depletion, and increasing antioxidant enzyme activities after submergence [51].

### 4.3. Molecular Mechanisms and Key Genes

The physiological responses of rice to flooding have a direct genetic and molecular basis, with specific loci and regulatory networks determining the type, extent, and success of the adaptive strategy. The SUBMERGENCE1 (SUB1) locus, particularly the SUB1A‐1 allele, is a key genetic breakthrough in rice flood tolerance. Identified in the Indian landrace FR13A, this QTL on chromosome 9 enables survival under complete submergence for 14 days [52]. Its quiescence strategy involves the ethylene‐responsive transcription factor SUB1A, which inhibits gibberellin‐driven shoot elongation, conserving energy and carbohydrates for postflood recovery [53]. As a transcriptional regulator, SUB1A suppresses gibberellin‐induced growth by enhancing the expression and stabilization of the DELLA repressors SLENDER RICE‐1 (SLR1) and SLR1‐like‐1 (SLRL1), thereby curbing excessive shoot elongation [35].

Deepwater rice varieties possess major QTLs on chromosome 12, closely associated with SNORKEL1 (SK1) and SNORKEL2 (SK2) genes, both ethylene responsive factor (ERF) transcription factors [19]. Unlike SUB1A, the SK1 and SK2 genes of deepwater rice are regulating the escape strategy [54]. Under deep flooding, ethylene strongly induces SK1 and SK2, which act as master regulators of the escape response by activating gibberellin biosynthesis and signaling pathways, therefore driving rapid internode elongation [19]. This fast stem growth ensures that rice foliage re‐establishes contact with the air, sustaining photosynthesis and plant survival in deep‐water conditions [19].

### 4.4. Limitations of the SUB1 Gene

The SUB1 locus is the primary genetic determinant of submergence tolerance in rice. Marker‐assisted backcrossing of the SUB1A‐1 haplotype of rice into mega‐varieties (Swarna‐Sub1, IR64‐Sub1, and NERICA‐Sub1) has transformed rice production in flood‐affected areas. These SUB1 varieties, which are widely used in South/Southeast Asia and Sub‐Saharan Africa, are characterized by the restriction of elongation, more rapid recovery, earlier flowering/maturity and grain quality maintenance during short‐term flooding, and a drastic reduction of otherwise disastrous yield losses [55].

**FIGURE 1:**
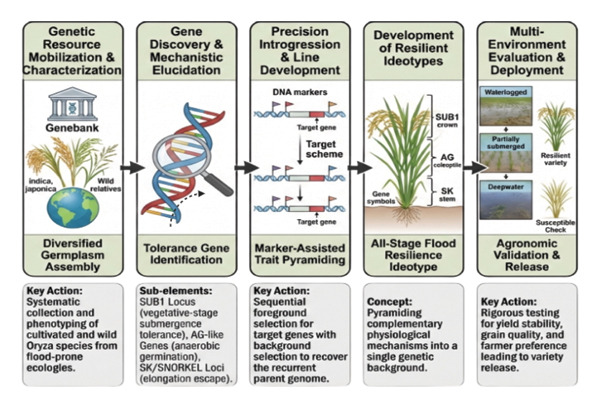
Strategic framework for the development of climate‐resilient rice varieties with enhanced flooding tolerance.

Although flood tolerance via SUB1A proved effective, it does have several limitations. It is only effective in the short‐term (< 10–14 days) flooding [56], and it does not work in cases of prolonged stagnant flooding, deepwater, or late flooding, where chronic adaptation is required [35]. Also, SUB1A does nothing to extend polygenic tolerance to a wide variety of agroecologies and does not offer oxygen‐starved germination tolerance in direct‐seeded crops [57] (Table 3). The breeding of the future should be able to shift beyond single gene solutions to reduce the impact of the entire range of flood stress typologies (Figure 1). As an example, it is possible to recombine SUB1A (short‐term submergence tolerance) and SNORKEL1/2 (deepwater adaptation) to make variants that tolerate both sudden flash floods and sustained inundation [56]. On the same note, the combination of SUB1A and AG1 (tolerance under AG) would confer resistance during germination to subsequent development, which is an indispensable characteristic in a direct‐grown rice system [55].

**TABLE 3:** Comparative analysis of rice survival strategies and the limitations of SUB1A.

Flooding type	Primary survival strategy	Role of *SUB1A*	Reason for limitations	Breeding and management implications
Germination stage oxygen deficiency (GSOD)	Anaerobic germination (AG)	None	*SUB1A* is not expressed during germination; lacks mechanisms for starch mobilization under anoxia	Requires AG1 or AG2 genes; use of seed priming
Flash flooding	Quiescence (energy conservation)	High	*SUB1A* suppresses GA‐mediated elongation, conserving carbohydrates for recovery	Highly effective for 10–14 days; widely deployed
Stagnant flooding	Moderate elongation and metabolic adaptation	Low	Quiescence strategy is maladaptive for long‐term (weeks/months) partial inundation	Needs genes for moderate elongation and nutrient use efficiency
Deep‐water flooding	Escape (rapid stem elongation)	Low (negative)	*SUB1A* inhibits the rapid elongation required to keep foliage above rising water	Requires SNORKEL1 and SNORKEL2 genes
Saline flooding	Ion exclusion and osmotic adjustment	None	*SUB1A* does not confer specific tolerance to Na + toxicity or osmotic stress	Requires pyramiding with salinity tolerance QTLs (e.g., Saltol)
Germination stage oxygen deficiency (GSOD)	Anaerobic germination (AG)	None	*SUB1A* is not expressed during germination; lacks mechanisms for starch mobilization under anoxia	Requires AG1 or AG2 genes; use of seed priming

## 5. External Factors Affecting Submergence Survival

Submergence is a multifaceted stress whose impact on plant survival is strongly mediated by external environmental factors during flooding and after water recession. Multiple studies demonstrate that floodwater physical characteristics, especially depth, turbidity, and light attenuation, are primary drivers of stress severity during submergence. Increased water depth consistently reduces the transmission of photosynthetically active radiation (PAR), limiting underwater photosynthesis and accelerating carbohydrate depletion [58–60]. High turbidity or shading significantly limits light penetration, reducing underwater photosynthesis and depleting carbohydrate reserves. Even highly tolerant cultivars exhibit increased mortality when exposed to turbid water, demonstrating that light limitation exacerbates submergence stress [61]. Light transmission through floodwater is further reduced by water depth, turbidity, sediment load, dissolved organic matter, intensifying shading, and suppressing photosynthesis. Water temperature plays also a critical role. Cooler floodwater reduces plant respiratory demands and increases oxygen solubility, thereby decreasing tissue anoxia and improving survival. In contrast, warmer floodwater accelerates carbohydrate depletion and reduces survival [58, 62, 63], with studies showing mortality increasing by approximately 8% for each degree rise above 26°C [61]. Floodwater chemistry also contributes to survival outcomes. Elevated carbon dioxide in turbid water can transiently enhance photosynthesis, while reduced oxygen availability limits metabolic activity. Water pH affects CO2 solubility and thus photosynthetic capacity, while low pH can improve CO2 availability and support survival under submerged conditions. Sediment load on plant surfaces, which increases with higher silt concentrations, further impairs growth and survival, likely through mechanical stress and interference with leaf gas exchange [61]. After cessation of submergence, the sudden re‐exposure to oxygen and light imposes an additional stress phase, characterized by oxidative stress, accelerated leaf senescence, and exposure of weakened tissues to dehydration and pathogens [64, 65].

## 6. Mitigation Strategies of Submergence Stress in Rice Production

### 6.1. Crop Management

#### 6.1.1. Use of Flood‐Tolerant Rice Varieties

The most successful stories about flood‐tolerant rice varieties are reported in Asia. Remarkable examples are the development and release of the flood‐tolerant varieties Swarna‐Sub1, Samba Mahsuri‐Sub1, IR64‐Sub1, CR1009‐Sub1, BR11‐Sub1, Ciherang‐Sub1, TDK1‐Sub1, and PSB Rc18‐Sub1 in India, Bangladesh, Myanmar, Nepal, and Indonesia. These SUB1 varieties showed no yield penalty under normal conditions compared to the non‐SUB1 varieties. However, they demonstrated an 80%–95% survival rate under flood conditions and provided up to a 3 t/ha yield gain compared to the intolerant varieties [66–70].

In Africa, the use of submergence‐tolerant rice varieties is still limited, particularly due to the absence of improved varieties carrying the Sub1 gene. However, research institutions like AfricaRice have been able to develop two varieties that are tolerant to submergence [59]. These varieties, namely FARO 66 and FARO 67, have been released in Nigeria in 2017. Under nonstress conditions, these flood‐tolerant rice varieties exhibit agronomic performance comparable to their recurrent parent lines; however, under submergence stress, they demonstrate a yield advantage of up to 1 ton per hectare [56, 71]. On‐farm evaluations of these varieties were conducted in many other countries of Africa (Madagascar, Ghana, Gambia, Sierra Leone, etc.) affected by flood events for participatory selection. The adoption of these varieties would significantly improve rice production in flood‐prone areas and contribute to ensuring food and nutritional security for rice farmers [72]. Furthermore, the introgression of the SUB1 gene into high‐yielding varieties of African countries through marker‐assisted backcrossing is highly recommended to develop submergence‐tolerant varieties adapted to local conditions and appreciated by local farmers [59].

In addition to the SUB1 gene, the SNORKEL genes (SK1 and SK2) have been identified and are known to confer tolerance to deep‐water stress in rice. The *SK1* and *SK2* genes enable rice plants to survive under deep water conditions by promoting stem elongation. During deep‐water stress, deep‐water–tolerant varieties exhibit rapid growth, extend their internodes, and keep their leaves above the water surface [19]. While SNORKEL genes allow rice varieties to survive in deep water, they do not enable rice to withstand prolonged submergence (weeks or months). Therefore, it is important to develop rice varieties harboring both SUB1 and SNORKEL genes capable of escaping during deep‐water stress at early growth stages and tolerating prolonged submergence at later stages.

Direct seeding is gaining importance in rice cultivation, especially because of the rise in transplanting costs. It has been demonstrated that traditional transplanting methods require 27 persons/days, whereas direct seeding with a drum seeder reduces labor demands to just 4 persons/days [73]. Direct seeding is, therefore, more cost‐effective and labor‐efficient and is also convenient for better crop establishment. Direct seeding methods comprise dry direct seeding (sowing of dry seed), wet direct seeding (sowing of pregerminated seeds), and water seeding (sowing of seed in water levels ranging from 5 to 15 cm) [74]. Direct seeding methods provide higher yield than transplanting methods [75]. Despite its advantages, direct seeding methods are highly susceptible to early‐season floods, which cause poor germination due to anaerobic conditions [76]. African farmers rely heavily on direct seeding, especially in rainfed lowlands rice fields of West and East Africa, and experience anaerobic stress caused by flooding [77]. It is, therefore, important to develop rice varieties with AG to mitigate the impact of early‐season flooding in African rice fields. AG tolerance depends on the action of a few genes and QTLs. The important genes are CIPK15 in the promotion of starch mobilization that enables the coleoptile to grow under oxygen deprivation [36] and the genes that code for fermentative substances such as PDC1 and ADH1, which are necessary to generate energy in the absence of oxygen [53]. Markers that can be used in enhancing AG include QTLs like qAG9.2 and qAG7.1 through marker‐assisted selection [56].

In Africa, many efforts have been undertaken to identify African rice germplasm with AG tolerance. Screening 2000 accessions of *Oryza glaberrima,* Agbeleye et al. [78] identified accessions TOG 5485, TOG 8347, TOG 16704, TOG 5505, and TOG 5980 as having higher survival rates during AG. Similarly, Asante et al. [79] identified another set of rice genotypes having more than a 70% survival rate under AG. Furthermore, the DANIDA‐funded project, Climate Smart African Rice, aiming at developing salinity‐ and flood‐tolerant rice varieties, has recently identified potential donors with anaerobic stress tolerance during germination [80]. The identification of these high‐survival genotypes provides valuable donor materials for breeding programs to enhance flood resilience.

Recently, genome editing technology, especially CRISPR‐Cas9 (clustered regularly interspaced short palindromic repeats/CRISPR‐associated protein 9), has transformed plant breeding as it has helped to achieve region‐specific changes at the genomic level in rice [81]. This technique allows the knock‐in, knock‐out, or alteration of flood‐sensitive genes and regulatory factors. As an example, CRISPR‐Cas9 may adjust the expression of ERF genes or optimize the promoter zone under the conditions of floods. In addition, it has the potential to transfer desirable alleles of LOES (low oxygen escape syndrome) from wild relatives to cultivated ones or turn off the S1 locus to overcome hybrid sterility in interspecific hybrids to allow an increase in the genetic base of flood tolerance [82]. Currently promising flood‐resilient rice lines are in development using CRISPR‐Cas9 technology (Table 4).

**TABLE 4:** CRISPR‐edited flood‐resilient rice lines in the pipeline (2020–2024).

Gene/target	Editing strategy	Required outcome	Status, year, country	Source
SUB1A promoter	Promoter modification	Enhanced expression under specific flood conditions	Research Phase, (2023), China, Colombia, and Philippines (IRRI)	[83, 84]
SK1/SK2	Gene knock‐in/overexpression	Optimized elongation for deepwater conditions	Field Trials, (2024), Philippines (IRRI), China	[83, 85]
AG1	Enhanced expression/allelic variation	Improved anaerobic germination	Pre‐Commercial, (2024), Philippines (IRRI), China	[83]
CIPK15	Overexpression	Increased starch mobilization for AG	Research Phase, (2023), Philippines (IRRI), China	[81, 84]
LBD37	Enhanced expression	Improved aerenchyma formation	Research Phase, (2022), Philippines (IRRI), China	[81, 84]
OsHAK1/OsSOS1	Enhanced expression	Increased tolerance to saline floods	Research Phase, (2024), Philippines (IRRI), China	[81, 85]
S1 locus	Gene knock‐out/suppression	Overcome hybrid sterility in interspecific crosses	Research Phase, (2025), Philippines (IRRI), China	[82, 85]

### 6.2. Seed and Seedling Management

Under direct seeding of rice in rainfed lowland areas, poor establishment of rice seedlings caused by submergence can be managed by the use of a higher seed rate than the recommended rate in normal conditions [86]. According to Illangakoon et al. [87] and Lal et al. [88], combining a high seed rate with AG‐tolerant rice varieties enhances seedling establishment by 13%–15% and boosts panicle density by up to 15%. Under the transplanting rice cultivation method, seedling age plays a significant role in ensuring a higher survival rate and yield when flooding occurs [89]. Studies have shown that older seedlings between 35 and 45 days produce higher yield than young seedlings under submergence conditions [62]. This is because older seedlings possess more biomass and greater carbohydrate content at transplanting and therefore exhibit a higher tolerance level [62].

Effective seed and seedling management is substantially greater when applied to genotypes carrying the SUB1 locus, particularly under severe or prolonged submergence stress. Seed pretreatments such as presoaking and hydropriming have been shown to enhance early emergence and seedling vigor under flooded conditions, but these benefits were strongly genotype‐dependent [90]. SUB1 genotypes showed limited protection during germination, whereas genotypes combining SUB1 with AG QTLs exhibited markedly higher emergence, shoot elongation above the water surface, biomass accumulation, and establishment under flooding [90]. Similarly, seed management for crop establishment through wet seeding increased early plant density and biomass prior to submergence and partially mitigated damage in non‐SUB1 genotypes, while SUB1 varieties consistently showed superior tolerance, faster post‐submergence recovery, and higher yields across establishment methods, indicating that management alone cannot substitute for genetic tolerance [59]. Seed priming through halopriming moderately has also been shown to improve physiological stability under submergence by reducing chlorophyll loss and maintaining photosystem II efficiency, with the submergence‐tolerant genotype consistently exhibiting higher shoot dry mass and relative growth rate than the non‐SUB1 genotype [91]. The effect of nursery management has also been studied. Banayo et al. [92] showed that management of nursery practices and seedling vigor through reduced seeding rate, transplanting older seedlings, and increased planting density did not significantly affect survival of SUB1 genotypes compared to non‐SUB1 genotypes but significantly enhanced carbohydrate reserves, post‐submergence recovery, and grain yield, while submergence‐intolerant genotypes failed to survive prolonged flooding, underscoring that seedling management strengthens but cannot replace SUB1‐mediated tolerance [92].

### 6.3. Nutrient Management

The soil fertility and mineral nutrient content play a significant role in the adaptation and recovery of rice during and after flood stress [93]. Specifically, application of nitrogen, phosphorus, and potassium before and after submergence stress could positively or negatively affect survival and recovery rate [86]. Across multiple studies, nitrogen applied shortly before submergence, typically within a few days to 1 week prior to flooding, has been shown to be deleterious, as it stimulated shoot elongation, increased succulence and metabolic demand, accelerated chlorophyll degradation, and caused rapid depletion of soluble sugars and starch, thereby reducing survival, particularly in non‐SUB1 genotypes [62, 65, 94]. Even in Sub1 cultivars, which inherently restrict elongation and conserve carbohydrates, pre‐submergence nitrogen failed to confer benefit and often intensified carbohydrate loss when flooding followed soon after application [65, 95]. In contrast, nitrogen applied after flood recession, typically 48 h to several days post‐desubmergence, was consistently beneficial, as it restored leaf nitrogen status, chlorophyll content, photosynthetic capacity, and biomass accumulation, contributing to accelerate recovery and improve survival and yield across genotypes, with the strongest and most stable gains observed in SUB1 genotypes [60, 63, 95, 96]. The physiological gap underlying these contrasting outcomes is explained by carbohydrate dynamics: Survival and productivity were more closely associated with nonstructural carbohydrate levels after desubmergence than before flooding, indicating that nitrogen is beneficial only when oxygen availability allows its assimilation to support regeneration rather than drive anaerobic energy depletion [62, 65, 97]. Basal phosphorus and potassium played critical complementary roles by enhancing seedling vigor, suppressing excessive elongation, stabilizing membranes, and conserving carbohydrates during submergence by creating the physiological capacity for effective postflood nitrogen use [64, 94]. In contrast, application of nitrogen in the nursery before flooding weakened vigor and survival by exhausting energy reserves.

### 6.4. Weather Forecast for Flood Prevision and Adaptive Decision‐Making

Weather forecasting could be strategically used for mitigating crop losses and enhancing preparedness and resilience among rice farmers. Forecast information, when accurate and timely, can guide farm‐level decisions across the cropping cycle, including sowing, transplanting, fertilization, irrigation, and harvest, thereby reducing the risk of damage from extreme weather events [98].

Weather and flood forecasts, when combined with adaptive cultivation practices, can substantially improve rice production by reducing submergence stress through anticipatory management. In flood‐dependent wetland systems such as the Inner Niger Delta in Mali, rice farmers actively use climate information on the onset of the rainy season, the timing of flood arrival, flood height, and flood duration to align rice establishment and growth with hydrological dynamics [99]. Forecast‐informed decisions allow farmers to adjust land preparation and sowing dates so that seedlings establish before inundation, avoiding early submergence, while delayed planting is used when late floods are predicted to prevent prolonged flooding during sensitive growth stages. Farmers further reduce submergence risk by reallocating cultivation along the floodplain gradient and selecting rice varieties with growth durations matched to expected flood length, thereby synchronizing crop phenology with flood recession. In contrast, in rainfed lowland rice systems of Northern Ghana, where submergence is driven mainly by intense rainfall and waterlogging rather than river flooding, seasonal and short‐term weather forecasts are used to adjust planting calendars, fertilizer application, and field operations to avoid heavy rainfall events that cause transient flooding and nutrient loss [100]. While Malian floodplain farmers emphasize spatial field positioning and varietal choice to cope with predictable inundation, Ghanaian farmers rely more on temporal adjustments of management practices to reduce rainfall‐induced submergence, reflecting differences in hydrological drivers and landscape constraints. Despite these contextual differences, both cultivation environments show that integrating weather forecasts with locally appropriate rice management reduces mismatches between crop growth stages and excess water exposure, lowers seedling mortality, stabilizes yields, and enhances resilience of African rice systems under increasing climate variability [99, 100].

However, the use of weather information among smallholder farmers remains limited due to gaps in communication infrastructure, forecast localization, and user trust. Climate services in agriculture face criticism by farmers for being overly coarse in scale, too technical for illiterate farmers, often inaccurate, and poorly communicated in terms of forecast probabilities [100]. To be fully effective and enable adaptive decision‐making by farmers, weather forecasts must take the reality of farmers into account by translating the information into local language and using community‐based radio to disseminate the information. Additionally, smallholder farmers typically possess limited risk‐bearing capacity and therefore require support to utilize forecast information that carries a high degree of probability and reliability. Furthermore, policies must be put in place to invest in national meteorological services, early warning systems, and timely delivery of information to farmers in their local language.

## 7. Flooding and Submergence Variability and Management Strategies in African Rice Systems

Flooding and submergence are recurrent events in African rice‐growing environments, but their nature varies substantially across regions in terms of origin, timing, duration, depth, and agronomic consequences. In the Inner Niger Delta and Middle Niger Basin of Mali and Niger, flooding is predominantly fluvial, originating from upstream rainfall and river discharge, and follows a relatively predictable seasonal pattern. Studies from this region indicate that rice production systems, including floodplain, pond, and deepwater rice, are adapted to long‐duration inundation, where adequate flood depth and timing are essential for optimal yields [99, 101]. However, excessively prolonged or unusually deep floods, such as Guinean floods lasting several weeks beyond normal recession periods, exceed varietal tolerance and lead to crop failure. In contrast, rice‐growing areas in Southern Mali, particularly Sikasso and the Segou/Mopti regions, experience both rainfed lowland and irrigated rice systems that are exposed to temporally distinct flood regimes. Mathieu et al. [102] demonstrate that early‐season flooding between June and August is largely agronomic and beneficial, facilitating land preparation and rice transplanting, whereas mid‐season flooding from September to November occurs during reproductive stages and causes severe yield losses in both rainfed and irrigated fields. This temporal differentiation is critical, as early floods dominate in Sikasso, while Segou and Niger Delta systems experience a higher proportion of damaging mid‐season floods, particularly in low‐lying fields close to the Niger River. In Eastern Africa, floodplain rice systems in the Kilombero and Lower Rufiji basins of Tanzania are primarily affected by riverine flooding driven by seasonal rainfall and upstream runoff, resulting in standing water and prolonged submergence during the main rainy season [12]. Unlike the Inner Niger Delta, flooding in these systems is less predictable and more frequently disrupts crop establishment and vegetative growth, especially under direct seeding, leading to AG failure and vegetative‐stage submergence. Coastal and deltaic environments, such as those in Benin and parts of the Niger Delta, are similarly characterized by prolonged inundation due to river overflow and poor drainage, where flood depth and duration during sensitive growth stages rather than flood onset itself determine yield outcomes [13]. Given this diversity, management of submergence across Africa relies more on adaptation and recovery than on flood avoidance. In flood‐dependent systems of the Inner Niger Delta, farmers adjust planting dates and spatial crop placement to synchronize crop development with flood rise and recession [99]. In submerged lowland systems of Mali’s Niger Delta, delayed planting until floodwaters recede is a common coping strategy [102], whereas in rainfed lowlands and floodplains of Tanzania and Benin, such flexibility is limited, increasing vulnerability to unpredictable floods. In these latter environments, the deployment of submergence‐tolerant rice varieties becomes particularly important [12, 13]. Flood risk mapping studies further emphasize the need for spatial targeting of stress‐tolerant varieties, recommending their deployment in areas prone to mid‐season and unpredictable flooding while avoiding zones where flood duration exceeds physiological tolerance thresholds [101, 102]. Therefore, flooding across African rice systems cannot be treated as a uniform stress. While some environments depend on predictable seasonal inundation for productivity, others experience damaging submergence that requires stress‐tolerant varieties and recovery‐focused agronomic interventions. Effective recommendations must therefore be context‐specific, integrating local flood regimes with appropriate combinations of planting strategies, nutrient management, spatial planning, and varietal selection. A blanket approach to flood management in African rice systems is inappropriate; instead, resilience depends on aligning cultivation technologies and stress‐tolerant varieties with the dominant flooding characteristics of each location.

## 8. Conclusion

Flooding imposes multiple, stage‐specific constraints on rice production, ranging from AG failure to complete submergence, stagnant flooding, and prolonged deep‐water inundation. This review demonstrates that the impact of flooding on rice is not uniform but is determined by the interaction between flood characteristics (depth, duration, and timing), plant developmental stage, and genotype. Rice survival relies on distinct adaptive strategies such as quiescence and escape strategy, each governed by specific physiological and molecular pathways. While the deployment of SUB1‐based varieties has substantially reduced yield losses under short‐term flash flooding, their limited effectiveness under stagnant, deep, or recurrent floods highlights a critical gap in current breeding strategies.

Future progress in flood‐resilient rice production will depend on moving beyond single‐trait solutions toward the deliberate integration of multiple tolerance mechanisms. Priority research directions include pyramiding SUB1 with AG and moderate elongation traits, optimizing antioxidant and recovery responses, and exploiting genome editing tools to fine‐tune regulatory networks controlling growth–survival trade‐offs. Equally important is the alignment of varietal improvement with location‐specific flood regimes, particularly in African rice systems where flooding patterns differ markedly across floodplains, rainfed lowlands, and coastal zones. Agronomic interventions, such as targeted nutrient management, seedling age optimization, and forecast‐based decision‐making, should be viewed as complementary to, rather than substitutes for, genetic tolerance.

Under projected climate change scenarios, flooding will become more frequent, intense, and unpredictable, amplifying risks to smallholder livelihoods and regional food security. Strengthening rice system resilience therefore requires an integrated approach that links climate‐responsive breeding, adaptive management, improved water infrastructure, and accessible climate information services. By explicitly matching flooding typologies with appropriate genetic and agronomic solutions, future research and development efforts can shift from reactive yield loss mitigation toward proactive climate adaptation, ensuring the long‐term sustainability of rice production in flood‐prone regions.

## Author Contributions

Abdoul‐Razak Oumarou Mahamane, Junior S. Kamara, Moise Hubert Byiringiro, and Alpha Sow conceptualized and wrote the original draft. Abdoul‐Razak Oumarou Mahamane critically reviewed and edited the manuscript.

## Funding

No funding was available for this work.

## Disclosure

All authors have read and approved the manuscript.

## Conflicts of Interest

The authors declare no conflicts of interest.

## Data Availability

There are no data associated with this work.
